# Examining the safety of mirabegron: an analysis of real-world pharmacovigilance data from the US FDA adverse event reporting system (FAERS) database

**DOI:** 10.3389/fphar.2024.1376535

**Published:** 2024-03-18

**Authors:** Junwei Wang, Aiwei Zhang, Miaoyong Ye, Cunming Zhang

**Affiliations:** ^1^ Department of Urology, Wenling Hospital Affiliated to Wenzhou Medical University (The First People’s Hospital of Wenling), Taizhou, Zhejiang, China; ^2^ Department of Ultrasound, Wenling Hospital Affiliated to Wenzhou Medical University (The First People’s Hospital of Wenling), Taizhou, Zhejiang, China

**Keywords:** mirabegron, overactive bladder, adverse event, pharmacovigilance, FAERS

## Abstract

**Background:** Mirabegron, the first β-3 adrenergic receptor agonist, received approval from the Food and Drug Administration (FDA) in 2012 for the treatment of overactive bladder (OAB). This pharmacovigilance study investigated the safety profile of mirabegron treatment using the US FDA Adverse Event Reporting System (FAERS) database.

**Methods:** This study employed disproportionality analyses, including the reporting odds ratio (ROR) and Bayesian Confidence Propagation Neural Network (BCPNN) algorithm, to quantify signals of adverse events associated with mirabegron.

**Results:** From the first quarter of 2012 to the third quarter of 2023, a comprehensive total of 14,356,234 adverse event (AE) reports were submitted to the FDA Adverse Event Reporting System database. Within this dataset, encompassing 18,763 reports specifically associated with mirabegron, healthcare professionals notably contributed 2,902 of these reports. A total of 80 preferred terms (PTs) of interest were identified using both the ROR and information component algorithms. The most common AEs included blood pressure increased, urinary retention, atrial fibrillation, dry mouth, and tachycardia, which were consistent with the product instructions. Unexpected significant AEs, such as arrhythmia, palpitations, dementia, transient ischemic attack, Parkinson’s disease, anti-neutrophil cytoplasmic antibody positive vasculitis, lip swelling, and swollen tongue, were also identified. The study findings indicated that the majority of onset time occurred within 30 days (n = 358, 55.68%). However, AEs were still possible after 1 year of mirabegron treatment.

**Conclusion:** This study provided valuable evidence for the real-world safety of mirabegron, helping clinical professionals enhance their understanding of mirabegron’s safety in clinical practice. It also contributed valuable evidence for further safety studies on mirabegron.

## Introduction

Overactive bladder (OAB) is a syndrome characterized by urgency as the core symptom, often accompanied by increased frequency of urination, nocturia, and with or without urge urinary incontinence (Haylen et al., 2016). OAB can have significant negative impact on the quality of life and impose a substantial socioeconomic burden (Lu et al., 2023). Preliminary studies suggest that among individuals aged 18 and older, the prevalence of overactive bladder (OAB) is 16.0% in males and 16.9% in females, respectively (Stewart et al., 2003). The primary approach to treating OAB initially focuses on non-pharmacological interventions, encompassing behavioral and educational measures like moderating caffeine and alcohol consumption, engaging in bladder training, and performing pelvic floor muscle exercises (Frey et al., 2023). The secondary treatment option entails pharmacotherapy, including the use of anticholinergic medications or β-3 adrenergic receptor agonists (Frey et al., 2023).

In 2012, mirabegron, a β-3 adrenergic receptor agonist, gained approval from the Food and Drug Administration (FDA) for treating OAB, inducing detrusor smooth muscle relaxation during the storage phase and increasing bladder capacity (Zuchowski et al., 2022; Kuo and Kuo, 2023). According to a Phase III, randomized, multicenter study (Herschorn et al., 2013), mirabegron is an effective treatment option for OAB, with a low incidence of adverse events (AEs). Compared to antimuscarinics, mirabegron demonstrates comparable efficacy; however, it exhibits fewer AEs (Sartori et al., 2023). Hence, mirabegron is being embraced and utilized more extensively in the management of OAB.

Based on drug instructions and randomized controlled studies, common AEs associated with mirabegron include nausea, headache, hypertension, constipation, dizziness, tachycardia, and nasopharyngitis (Nitti et al., 2013a; Herschorn et al., 2013; Yamaguchi et al., 2015). However, some new and uncommon AEs have gradually been identified, such as tongue angioedema, serum sickness-like reaction, and respiratory dysfunction (Malsin et al., 2019; Tan et al., 2019; Zuchowski et al., 2022). As the use of mirabegron becomes more widespread, it is crucial to heighten awareness of its safety. Particularly notable are AEs not explicitly mentioned in the drug instructions, as they may be overlooked by both clinicians and patients during the course of medication. Therefore, this study aimed to evaluate signals of AEs associated with mirabegron.

The US Food and Drug Administration (FDA) Adverse Event Reporting System (FAERS) is a spontaneous reporting system (SRS) used to assess potential associations between drugs and AEs (Sharma and Kumar, 2022; Shu et al., 2022). Disproportionality analysis is a common method used to detect signals in drug vigilance databases and is employed to identify unknown AEs (Noguchi et al., 2021; Javed and Kumar, 2024). The reporting odds ratio (ROR) is a method of frequency disproportionality analysis, while the information component (IC) is a component of Bayesian disproportionality analysis (Ji et al., 2021). ROR is a classic method and also an algorithm frequently used by the Pharmaceuticals and Medical Devices Agency of Japan (PMDA) and the Netherlands Pharmacovigilance Centre (Lareb) (Noguchi et al., 2021). Research indicated that ROR was more advantageous than the proportional reporting ratio (PRR) in spontaneous reporting databases (Rothman et al., 2004). The advantage of IC lies in penalizing small-sample signals, thereby reducing the likelihood of chance findings and improving the reliability of analysis results, especially when sample sizes are small (Ji et al., 2021). This study, based on the FAERS, investigated the safety profile of mirabegron in the post-market setting using the ROR and IC methods.

## Materials and methods

### Data source and study design

The data for this study originated from the FAERS, which supports the FDA’s post-market surveillance programs for all marketed drugs and therapeutic biologics. It is a large-scale pharmacovigilance database that encompasses seven major datasets: demographics, drugs, reactions, indications, therapies, outcomes, and report sources. The reported information is provided by healthcare professionals such as physicians (MD), pharmacists (PH), and other health-professional (OT), as well as consumers such as patients, family members, and lawyers. To ensure the reliability of reporting sources, this study extracted and analyzed reports submitted by healthcare professionals. FAERS classifies reported drugs into four categories: PS (Primary Suspect), SS (Secondary Suspect), C (Concomitant), and I (Interacting). AEs and medication errors are coded using terms in the Medical Dictionary for Regulatory Activities (MedDRA), which is a comprehensive and detailed standard medical terminology developed by the International Council for Harmonisation of Technical Requirements for Pharmaceuticals for Human Use (ICH). MedDRA provides a five-level structure, including system organ class (SOC), high-level group term (HLGT), high-level term (HLT), preferred term (PT), and lowest level term (LLT). Mirabegron was approved by the FDA for the treatment of OAB in 2012. Data from the FAERS database, encompassing the initial quarter of 2012 through the third quarter of 2023, was acquired. In this study, both the generic and brand names such as Betmiga, Betanis, Myrbetriq, and Mirabegron were used for retrieval. Furthermore, only reports provided by healthcare professionals and reports exclusively documenting mirabegron as the PS drug were included in our analysis. For duplicate reports, we conducted deduplication based on the method recommended by the FDA (Zhang et al., 2023). Firstly, the PRIMARYID, CASEID, and FDA_DT fields were selected from the DEMO table and sorted in ascending order based on CASEID, FDA_DT, and PRIMARYID. For reports with the same CASEID, the one with the maximum FDA_DT value was retained. Subsequently, for reports with identical CASEID and FDA_DT values, the one with the maximum PRIMARYID value was retained. At the SOC level, we excluded AEs such as “injury, poisoning and procedural complications”, “product issues”, “surgical and medical procedures”, and “social circumstances”, which were unrelated to drug-related AEs. Finally, we obtained information including age, weight, gender, indications, drug usage, treatment outcomes, the start date of treatment, the occurrence date of adverse events, etc. The time-to-onset of AEs was defined as the period from the start date of treatment to the date of AE occurrence. Critical patient outcomes were outlined as hospitalization-initial or prolonged (HO), death (DE), disability (DS), life-threatening (LT), congenital anomaly (CA) or other important medical event (OT).

### Statistical analysis

In this study, we categorized age into five groups (≤17 years, 18–64 years, 65–85 years, ≥86 years, unknown), and weight into four groups (<50 kg, 50–100 kg, >100 kg, unknown). We employed descriptive analysis to highlight the clinical features found in AE reports associated with mirabegron. Disproportionality analysis stands as a frequently utilized method in pharmacovigilance. This study employed two approaches, reporting odds ratio (ROR) and Bayesian Confidence Propagation Neural Network (BCPNN), for detecting AE signals. The calculation formulas and positive safety signal thresholds were provided in Table 1. Here, “a” represents the number of target drug-specific adverse reactions, “b” represents other adverse reactions related to the target drug, “c” represents adverse reactions related to other drugs but involving the target drug, and “d” represents other adverse reactions not related to the target drug. In this study, we focused on AEs meeting both algorithm criteria for further investigation. All analyses were performed using R software version 4.3.2 (R Foundation for Statistical Computing, Vienna, Austria).

**TABLE 1:** Two major algorithms used to assess potential associations between mirabegron and adverse events.

Algorithms	Equation	Equation
ROR	ROR=ad/b/c	lower limit of 95% CI > 1, N ≥ 3
	$95\%\text{CI}=e^{\ln\left(\text{ROR}\right)\pm 1.96(1/a+1/b+1/c+1/d\hat{)}0.5}$	
BCPNN	$\text{IC}=\log_{2}a\left(a+b+c+d\right)/\left(\left(a+c\right)\left(a+b\right)\right)$	IC025 > 0
	$95\%\text{CI}=E\left(\text{IC}\right)\;\pm \;2V(\text{IC}\hat{)}0.5$	

Notes: Equation: a, number of reports containing both the target drug and target adverse drug reaction; b, number of reports containing other adverse drug reaction of the target drug; c, number of reports containing the target adverse drug reaction of other drugs; d, number of reports containing other drugs and other adverse drug reactions.

Abbreviations: ROR, reporting odds ratio; BCPNN, bayesian confidence propagation neural network; 95% CI, 95% confidence interval; N, the number of reports; IC, information component; IC025, the lower limit of 95% CI, of the IC.

## Results

### Population characteristics

**FIGURE 1:**
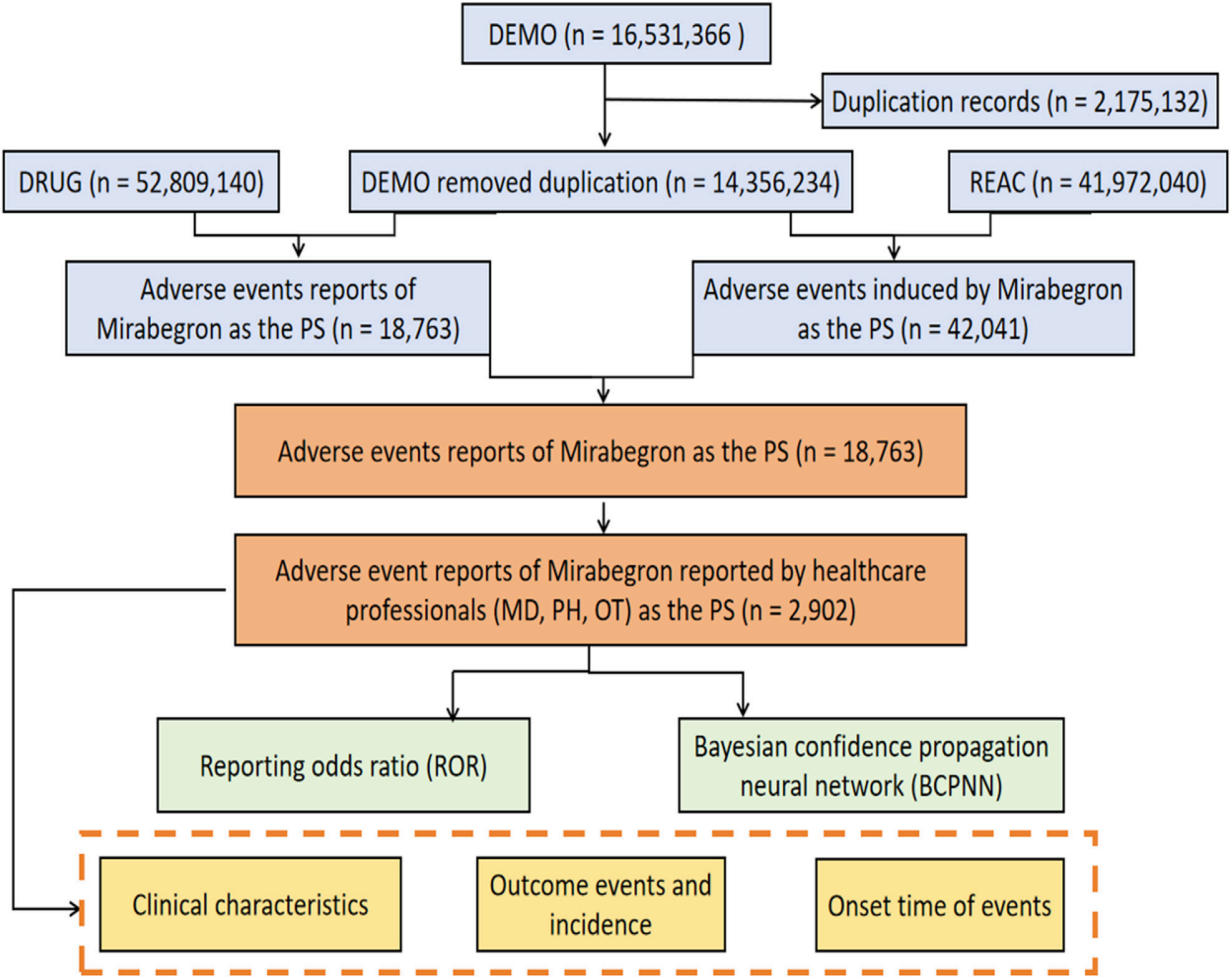
The process of searching mirabegron-associated adverse events from the US Food and Drug Administration (FDA) Adverse Event Reporting System (FAERS).

From the first quarter of 2012 to the third quarter of 2023, a total of 14,356,234 AE reports were submitted to the FAERS database, including 18,763 reports related to mirabegron. Among these, 2,902 reports were reported by healthcare professionals (Figure 1). Characteristics of the AE reports for mirabegron were summarized in Table 2. Female patients were more commonly reported than male patients (60.06% vs 34.77%). Elderly patients (age ≥65) accounted for the majority of AE reports (42.83%), excluding unknown reports. The United States had the highest reporting rate (49.52%). Excluding reports with unknown indications, hypertonic bladder (31.05%) and incontinence (13.71%) were the most common indications. Other medical events (51.00%) were the most frequently reported serious outcomes, followed by 490 cases of hospitalization (16.88%).

**TABLE 2:** Clinical characteristics of reports with mirabegron from the Food and Drug Administration (FDA) Adverse Event Reporting System (FAERS) database.

Characteristics	Case number, n	Case proportion, %
Number of reports	2902	
Sex		
Female	1743	60.06
Male	1009	34.77
Unknown	150	5.17
Weight,kg		
<50	38	1.31
50–100	216	7.44
>100	57	1.96
Unknown	2591	89.28
Age,year		
≤17	12	0.41
18–64	389	13.40
65–85	1055	36.35
≥86	188	6.48
Unknown	1258	43.35
Reported person		
Physician (MD)	1482	51.07
Other health-professional (OT)	1010	34.80
Pharmacist (PH)	410	14.13
Reporting country		
United States	1437	49.52
Japan	459	15.82
Great Britain	322	11.10
Spain	106	3.65
Others	578	19.92
Continent		
North America	1498	51.62
Europe	845	29.12
Asia	516	17.78
South America	12	0.41
Oceania	8	0.28
Africa	7	0.24
Unknown	16	0.55
Indication		
Hypertonic bladder	901	31.05
Incontinence	398	13.71
Pollakiuria	112	3.86
Micturition urgency	74	2.55
Nocturia	31	1.07
Neurogenic bladder	23	0.79
Benign prostatic hyperplasia	22	0.76
Outcome		
Death (DE)	54	1.86
Life-Threatening (LT)	65	2.24
Hospitalization - Initial or Prolonged (HO)	490	16.88
Disability (DS)	72	2.48
Congenital Anomaly (CA)	0	0
Other Serious (Important Medical Event) (OT)	1480	51.00
Drug dosage, mg/day		
12.5 mg	2	0.07
25 mg	149	5.13
25–50 mg	3	0.10
50 mg	289	9.96
75 mg	1	0.03
100 mg	7	0.24
150 mg	2	0.07
Unknown	2450	84.42
Duration of medication, days		
0–30	400	13.78
31–60	104	3.58
61–90	61	2.10
91–180	73	2.52
181–360	41	1.41
>361	50	1.72
Unknown	2173	74.88

### Signal detection

Mirabegron related AE reports involved 21 organ systems, and the signal strength at the SOC level was visible in Table 3. The most significant SOC was “renal and urinary disorders,” with positive reactions in both the ROR and information component (IC) methods. Signal detection for “nervous system disorders”, “cardiac disorders”, “eye disorders”, “reproductive system and breast disorders”, and “vascular disorders” showed positive results in the ROR method, while there was no positive signal in the IC method, suggesting that these signals may have also been important and frequent.

**TABLE 3:** Signal strength of AEs of mirabegron at the system organ class (SOC) level in FAERS database.

System organ class (SOC)	N	ROR (95%Cl)	IC(IC025)
General disorders and administration site conditions	817	0.81 (0.75–0.87)	−0.27 (-1.93)
Nervous system disorders	610	1.23 (1.13–1.33)	0.27 (-1.4)
Gastrointestinal disorders	558	1.07 (0.98–1.17)	0.09 (-1.58)
Cardiac disorders	506	2.63 (2.4–2.88)	1.32 (-0.35)
Renal and urinary disorders	445	3.56 (3.23–3.92)	1.76 (0.09)
Investigations	434	1.1 (1–1.21)	0.13 (-1.54)
Skin and subcutaneous tissue disorders	310	0.97 (0.86–1.08)	−0.05 (-1.71)
Vascular disorders	262	1.82 (1.61–2.06)	0.84 (-0.83)
Psychiatric disorders	240	0.83 (0.73–0.94)	−0.26 (-1.92)
Musculoskeletal and connective tissue disorders	213	0.71 (0.62–0.81)	−0.47 (-2.14)
Infections and infestations	201	0.52 (0.45–0.6)	−0.9 (-2.57)
Respiratory, thoracic and mediastinal disorders	197	0.61 (0.53–0.7)	−0.69 (-2.35)
Eye disorders	150	1.36 (1.16–1.6)	0.44 (-1.23)
Metabolism and nutrition disorders	76	0.47 (0.37–0.59)	−1.07 (-2.74)
Reproductive system and breast disorders	72	1.83 (1.45–2.31)	0.87 (-0.8)
Neoplasms benign, malignant and unspecified (incl cysts and polyps)	48	0.24 (0.18–0.32)	−2.03 (-3.7)
Immune system disorders	45	0.52 (0.39–0.69)	−0.94 (-2.61)
Hepatobiliary disorders	44	0.52 (0.39–0.7)	−0.94 (-2.6)
Ear and labyrinth disorders	26	1.18 (0.8–1.73)	0.23 (-1.43)
Blood and lymphatic system disorders	22	0.13 (0.08–0.19)	−2.95 (-4.62)
Endocrine disorders	8	0.4 (0.2–0.79)	−1.33 (-3)

As shown in Table 4, which described a total of 80 PTs of interest identified with both ROR and IC algorithms, among which 23 PTs were consistent with product instructions and warnings, including cystitis, acute pyelonephritis, rhinitis, blood pressure increased, head discomfort, dry throat, dry mouth, bowel movement irregularity, blood pressure systolic increased, urinary retention, dysuria, interstitial cystitis, blood pressure abnormal, aemorrhagic cystitis, atrial fibrillation, tachycardia, atrial tachycardia, hypertension, hypertensive crisis, accelerated hypertension, malignant hypertension, essential hypertension, dry eye. Blood pressure increased, urinary retention, hypertension, atrial fibrillation, dry mouth, and tachycardia were the most common AE reports.

**TABLE 4:** Signal strength of reports of mirabegron at the perferred terms (PTs) level in FAERs database.

SOC	PT	N	ROR (95%Cl)	IC(IC025)	Expected
Infections and infestations	Cystitis	15	4.93 (2.97–8.19)	2.3 (0.63)	Yes
	Pyelonephritis acute	3	8.3 (2.67–25.8)	3.05 (1.38)	Yes
	Rhinitis	3	3.51 (1.13–10.91)	1.81 (0.14)	Yes
Investigations	Blood pressure increased	166	14.72 (12.61–17.18)	3.84 (2.17)	Yes
	Electrocardiogram QT prolonged	28	4.26 (2.94–6.18)	2.08 (0.42)	No
	Heart rate irregular	13	8.26 (4.79–14.25)	3.04 (1.37)	No
	Blood pressure systolic increased	11	7.4 (4.09–13.38)	2.88 (1.22)	Yes
	Intraocular pressure increased	7	5.55 (2.64–11.66)	2.47 (0.8)	No
	Blood urine present	6	3.83 (1.72–8.54)	1.94 (0.27)	No
	Antineutrophil cytoplasmic antibody increased	5	153.88 (62.45–379.13)	7.18 (5.49)	No
	Blood pressure abnormal	5	3.52 (1.47–8.48)	1.82 (0.15)	Yes
Nervous system disorders	Dementia	17	7.27 (4.51–11.71)	2.85 (1.19)	No
	Transient ischaemic attack	17	4.35 (2.7–7.01)	2.12 (0.45)	No
	Parkinson’s disease	11	7.06 (3.91–12.77)	2.81 (1.15)	No
	Head discomfort	4	3.41 (1.28–9.08)	1.77 (0.1)	Yes
	Myasthenia gravis	3	4.1 (1.32–12.72)	2.03 (0.37)	No
Musculoskeletal and connective tissue disorders	Muscle tightness	4	3.22 (1.21–8.58)	1.68 (0.02)	No
	Muscle atrophy	3	3.4 (1.09–10.55)	1.76 (0.1)	No
Respiratory, thoracic and mediastinal disorders	Dry throat	5	8.21 (3.41–19.76)	3.03 (1.37)	Yes
	Hyperventilation	3	4.56 (1.47–14.16)	2.19 (0.52)	No
Psychiatric disorders	Hallucination, visual	8	3.39 (1.7–6.79)	1.76 (0.09)	No
	Abnormal dreams	5	3.78 (1.57–9.08)	1.91 (0.25)	No
	Apathy	5	4.55 (1.89–10.94)	2.18 (0.52)	No
	Eating disorder	5	3.36 (1.4–8.09)	1.75 (0.08)	No
Immune system disorders	Anti-neutrophil cytoplasmic antibody positive vasculitis	3	9.79 (3.15–30.43)	3.29 (1.62)	No
Skin and subcutaneous tissue disorders	Petechiae	6	4.19 (1.88–9.34)	2.06 (0.4)	No
General disorders and administration site conditions	Generalised oedema	8	5.04 (2.52–10.09)	2.33 (0.66)	No
	Thirst	5	4.04 (1.68–9.73)	2.01 (0.35)	No
Renal and urinary disorders	Urinary retention	128	31.97 (26.81–38.12)	4.95 (3.29)	Yes
	Dysuria	33	9.84 (6.98–13.86)	3.29 (1.62)	Yes
	Nocturia	29	33.45 (23.17–48.28)	5.04 (3.37)	No
	Urinary incontinence	29	10.63 (7.37–15.32)	3.4 (1.73)	No
	Pollakiuria	25	8.54 (5.76–12.66)	3.09 (1.42)	No
	Haematuria	22	4.24 (2.79–6.44)	2.08 (0.41)	No
	Micturition urgency	16	17.84 (10.91–29.19)	4.14 (2.48)	No
	Bladder pain	10	51.14 (27.34–95.65)	5.65 (3.98)	No
	Hypertonic bladder	7	29.52 (14.01–62.21)	4.87 (3.2)	No
	Urine flow decreased	5	33.55 (13.88–81.08)	5.05 (3.38)	No
	Postrenal failure	4	39.22 (14.61–105.29)	5.27 (3.6)	No
	Cystitis interstitial	4	35.11 (13.09–94.18)	5.11 (3.44)	Yes
	Cystitis haemorrhagic	4	5.56 (2.08–14.83)	2.47 (0.8)	Yes
	Hydronephrosis	4	3.44 (1.29–9.18)	1.78 (0.11)	No
	Bladder spasm	3	20.07 (6.45–62.52)	4.32 (2.64)	No
	Neurogenic bladder	3	11.57 (3.72–35.97)	3.53 (1.86)	No
	Urge incontinence	3	28.99 (9.29–90.46)	4.84 (3.17)	No
Reproductive system and breast disorders	Erectile dysfunction	12	6.25 (3.55–11.03)	2.64 (0.97)	No
	Benign prostatic hyperplasia	4	7.6 (2.85–20.3)	2.92 (1.25)	No
	Vulvovaginal discomfort	3	12.09 (3.89–37.6)	3.59 (1.92)	No
Gastrointestinal disorders	Dry mouth	56	10.48 (8.05–13.64)	3.37 (1.71)	Yes
	Lip swelling	19	5.17 (3.3–8.12)	2.37 (0.7)	No
	Swollen tongue	18	5.41 (3.41–8.6)	2.43 (0.76)	No
	Mouth swelling	6	10.14 (4.55–22.61)	3.34 (1.67)	No
	Peptic ulcer	4	9.07 (3.4–24.2)	3.18 (1.51)	No
	Bowel movement irregularity	4	8.23 (3.08–21.97)	3.04 (1.37)	Yes
	Colitis ischaemic	4	3.78 (1.42–10.09)	1.92 (0.25)	No
	Cheilitis	3	5.4 (1.74–16.78)	2.43 (0.76)	No
	Lip disorder	3	21.41 (6.87–66.71)	4.41 (2.74)	No
	Lip oedema	3	3.29 (1.06–10.2)	1.72 (0.05)	No
Cardiac disorders	Atrial fibrillation	91	7.44 (6.04–9.15)	2.87 (1.21)	Yes
	Arrhythmia	83	16.23 (13.06–20.17)	3.99 (2.33)	No
	Palpitations	66	6.74 (5.29–8.6)	2.74 (1.07)	No
	Tachycardia	53	4.19 (3.2–5.5)	2.06 (0.39)	Yes
	Angina pectoris	17	5.13 (3.19–8.26)	2.35 (0.69)	No
	Extrasystoles	8	12.09 (6.03–24.22)	3.59 (1.92)	No
	Atrial flutter	7	5.62 (2.68–11.81)	2.49 (0.82)	No
	Ventricular extrasystoles	7	5.47 (2.61–11.5)	2.45 (0.78)	No
	Sinus node dysfunction	5	9.45 (3.93–22.76)	3.24 (1.57)	No
	Cardiac fibrillation	4	31.59 (11.78–84.68)	4.96 (3.29)	No
	Cardiac discomfort	3	20.02 (6.43–62.37)	4.31 (2.64)	No
	Atrial tachycardia	3	9.94 (3.2–30.89)	3.31 (1.64)	Yes
Vascular disorders	Hypertension	121	5.93 (4.96–7.11)	2.54 (0.88)	Yes
	Hypertensive crisis	34	16.94 (12.08–23.75)	4.07 (2.4)	Yes
	Vasculitis	6	3.92 (1.76–8.73)	1.97 (0.3)	No
	Malignant hypertension	5	36.78 (15.21–88.92)	5.18 (3.51)	Yes
	Accelerated hypertension	5	91.07 (37.34–222.14)	6.46 (4.78)	Yes
	Essential hypertension	3	9.05 (2.91–28.13)	3.17 (1.5)	Yes
Eye disorders	Dry eye	15	4 (2.41–6.65)	2 (0.33)	Yes
	Eyelid oedema	10	6.09 (3.27–11.33)	2.6 (0.94)	No
	Glaucoma	7	4.76 (2.27–10)	2.25 (0.58)	No
	Retinal vein occlusion	5	11.96 (4.97–28.81)	3.57 (1.91)	No

A total of 57 PTs were unexpected findings of significant AEs. Among them, AE reports with a count exceeding 10 included arrhythmia, palpitations, nocturia, urinary incontinence, electrocardiogram QT prolonged, pollakiuria, hematuria, lip swelling, swollen tongue, dementia, angina pectoris, transient ischemic attack, micturition urgency, heart rate irregular, erectile dysfunction, Parkinson’s disease, bladder pain, and eyelid edema. Some PTs with elevated signal intensity were discovered, including antineutrophil cytoplasmic antibody increased (ROR = 153.88), bladder pain (ROR = 51.14), postrenal failure (ROR = 39.22), urine flow decreased (ROR = 33.55), nocturia (ROR = 33.45), cardiac fibrillation (ROR = 31.59), hypertonic bladder (ROR = 29.52), urge incontinence (ROR = 28.99), lip disorder (ROR = 21.41), bladder spasm (ROR = 20.07), and cardiac discomfort (ROR = 20.02).

### Subgroup analyses

In this study, we employed subgroup analysis to conduct further investigation. We investigated the relationship between weight and AEs sorted by signal intensity (Supplementary Figure S1). For the weight group <50 kg, two PTs were identified: urinary retention and tachycardia. For the 50–100 kg weight group, the top 10 PTs by signal intensity were cystitis haemorrhagic, muscle atrophy, micturition urgency, muscle tightness, heart rate irregular, kidney infection, cystitis, arrhythmia, urinary retention, and dysuria. For the >100 kg weight group, six PTs were identified: nocturia, urinary retention, urinary incontinence, atrial fibrillation, hypertension, and angioedema. Additionally, we explored AEs in North America, Europe, and Asia (Supplementary Figure S2). In the North American group, the top 10 PTs by signal intensity were: antineutrophil cytoplasmic antibody increased, urge incontinence, urinary retention, nocturia, bladder pain, urine flow decreased, cystitis interstitial, bladder spasm, hypertensive crisis, and micturition urgency. In the European group, the top 10 PTs were accelerated hypertension, bladder pain, malignant hypertension, hypertonic bladder, essential hypertension, cardiac fibrillation, cardiac discomfort, heart rate irregular, micturition urgency, and mouth swelling. In the Asian group, the top 10 PTs were peptic ulcer, arrhythmia, erectile dysfunction, postrenal failure, urinary retention, benign prostatic hyperplasia, retinal vein occlusion, dysuria, sinus node dysfunction, and dry mouth.

This study revealed that the top three drugs most commonly prescribed in conjunction with mirabegron were aspirin (163 cases), atorvastatin (122 cases), and solifenacin (112 cases). Subsequently, we conducted subgroup analysis of the combined drugs. In the group of mirabegron combined with aspirin, the top 10 PTs included ventricular extrasystoles, urinary retention, atrial fibrillation, palpitations, swollen tongue, tachycardia, blood pressure increased, dry mouth, haematuria, and acute myocardial infarction (Supplementary Figure S3). In the group of mirabegron combined with atorvastatin, the top 10 PTs included erectile dysfunction, heart rate irregular, urinary retention, atrial fibrillation, hyperglycaemia, palpitations, electrocardiogram QT prolonged, blood pressure increased, vision blurred, and visual impairment (Supplementary Figure S4). In the group of mirabegron combined with solifenacin, the top 10 PTs included dry mouth, urinary retention, dry eye, palpitations, acute myocardial infarction, delirium, constipation, visual impairment, atrial fibrillation, tachycardia (Supplementary Figure S5).

### Time-to-onset analysis

**FIGURE 2:**
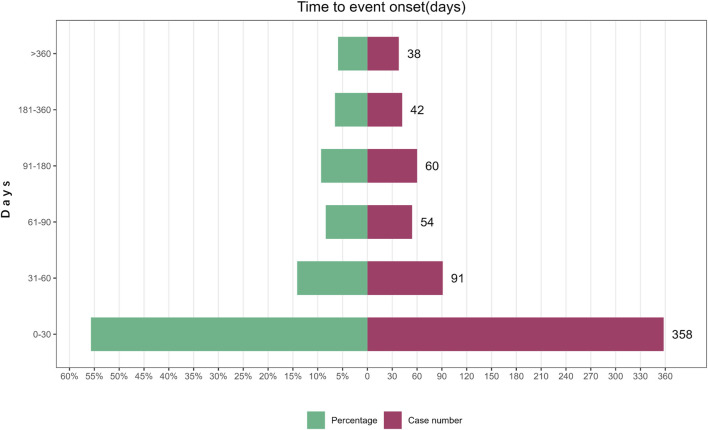
Time to onset of mirabegron-related AEs.

A total of 643 AE reports related to Mirabegron reported onset time, with a median onset time of 25 days (interquartile range [IQR] 7–75 days). The study findings indicated that the majority of onset time occurred within 30 days (n = 358, 55.68%). However, AEs were still possible after 1 year of mirabegron treatment, accounting for a proportion of 5.91% (Figure 2).

## Discussion

The FAERS is a spontaneous reporting system for AEs, providing a public database for the real-world assessment of the post-marketing safety profile of mirabegron. It enables the identification of AEs not yet documented in the drug instructions. To ensure the reliability of reporting sources, our analysis exclusively included reports provided by healthcare professionals and reports specifically documenting mirabegron as the PS drug. The incidence of AEs related to mirabegron were higher in females (60.10%) than in males (34.80%), excluding cases with unknown gender. This may be related to the higher prevalence of OAB in females compared to males (Xiao et al., 2023). Furthermore, with age increases, the prevalence of OAB increased gradually (Song et al., 2023). This phenomenon also explained the higher incidence of AEs among individuals aged 65 and older in this study. With the continuous expansion of mirabegron’s clinical application, clinicians should be vigilant about AEs associated with mirabegron, especially in elderly patients. Early identification of AEs is essential because these AEs may not only increase the likelihood of patient hospitalization but also pose potentially life-threatening risks.

Based on the disproportional analysis, the most frequent and significant signals at the SOC level were renal and urinary disorders, showing positive reactions in both the ROR and IC methods. The related AEs included dysuria, pollakiuria, bladder pain, bladder spasm, neurogenic bladder, etc., but they were often considered as indicative of mirabegron. Therefore, these AEs are considered to be associated with the progression of the underlying medical condition rather than being AEs caused by the medication itself. It is noteworthy that there were 128 reports of urinary retention, with significant signals observed in both the ROR and IC analyses. According to previous research, mirabegron did not have AEs affecting urinary urodynamic parameters, including maximum urinary flow rate and detrusor pressure at maximum flow, and did not increase the risk of urinary retention (Nitti et al., 2013b; Nitti et al., 2013c; Herschorn et al., 2013; Yamaguchi et al., 2015). Furthermore, the mechanism of mirabegron involves promoting relaxation of the detrusor muscle and increasing urinary storage capacity without altering voiding pressure or contraction pressure (Nitti et al., 2013a). Based on previous research findings, activation of the β3-adrenergic receptor through the exchange protein directly activated by cAMP (EPAC) pathway led to an increased release of adenosine, subsequently inhibiting the release of acetylcholine (ACh) in the bladder (Silva et al., 2017; Kuo, 2022). Mirabegron appeared to exhibit good safety regarding urinary retention. Therefore, a reasonable interpretation of the study results was that disease progression, such as benign prostatic hyperplasia or neurogenic bladder, might have been a contributing factor to urinary retention. However, according to a Japanese study, nineteen OAB patients with concomitant benign prostatic hyperplasia experienced urinary retention after using mirabegron, with resolution or recovery observed upon discontinuation (Takahashi et al., 2021). Meanwhile, according to a study, it was found that *in vitro*, obese mice fed with a high-fat diet released adenosine, which promoted proliferation of human prostate epithelial cell lines (Passos et al., 2023). Therefore, it is worth further investigating whether the increased adenosine release induced by mirabegron also indirectly leads to proliferation of prostate epithelial cells, thus affecting the occurrence of urinary difficulties or urinary retention. Therefore, we believe that the relationship between mirabegron and the AE of urinary retention remains controversial. For patients with evident bladder outlet obstruction in clinical practice, the use of mirabegron should be approached with caution (Takahashi et al., 2021). Additionally, we recommend regular follow-up during mirabegron treatment, during which clinicians should monitor patients’ voiding patterns, residual urine volume, and urinary flow rates.

Furthermore, significant signals were observed in the ROR method within the SOC categories of cardiac disorders, investigations, and vascular disorders. Based on findings from clinical studies, the predominant cardiovascular AEs linked to mirabegron include hypertension, tachycardia, palpitations, and atrial fibrillation (Nitti et al., 2013a; Batista et al., 2015; Rosa et al., 2016; Chan et al., 2022). Approximately 9%–10% of patients undergoing mirabegron treatment may experience hypertension (Krhut et al., 2021). Furthermore, there have been reports of an increase in the QTc interval (Balachandran and Duckett, 2015). In accordance with the results of this study, the ROR for blood pressure increased as 14.72 (12.61–17.18), with an IC of 3.84 (2.17). The ROR for hypertension as 5.93 (4.96–7.11), with an IC of 2.54 (0.88). For electrocardiogram QT prolonged, the ROR was 4.26 (2.94–6.18) with an IC of 2.08 (0.42). The ROR for atrial fibrillation was 7.44 (6.04–9.15) with an IC of 2.87 (1.21). Arrhythmia had an ROR of 16.23 (13.06–20.17) with an IC of 3.99 (2.33). The ROR for palpitations was 6.74 (5.29–8.6) with an IC of 2.74 (1.07), and for tachycardia, the ROR was 4.19 (3.2–5.5) with an IC of 2.06 (0.39). As mirabegron is a β3-adrenergic receptor agonist, and β3-adrenergic receptors are also expressed in cardiovascular tissues, the use of mirabegron may have “off-target” effects on the regulation of the cardiovascular system (Rosa et al., 2016). This could be the potential pharmacological mechanism by which mirabegron induces cardiovascular-related AEs. β3-adrenergic receptors can increase the occurrence of arrhythmias and the risk of atrial fibrillation by activating the cAMP-dependent protein kinase pathway and inducing a Ca2+ imbalance (Chan et al., 2022). Additionally, the β1-adrenergic receptors expressed in cardiovascular tissues were also target sites for the action of mirabegron (van Gelderen et al., 2017). An animal study found that the heart rate effects of mirabegron in dogs were attributed to its cross-reactivity with β1-adrenergic receptors (Korstanje et al., 2017). This was further confirmed by a clinical study (van Gelderen et al., 2017). Regardless of whether it involved the stimulation of β1-adrenergic receptors in cardiovascular tissues or β3-adrenergic receptors, the administration of supratherapeutic dosages that led to significant multiples of peak plasma concentrations was more prone to causing increases in heart rate, elevated blood pressure, and prolonged QT intervals (Malik et al., 2012; van Gelderen et al., 2017). Although a treatment dose of 50 mg mirabegron could lead to an increase in baseline pulse rate by one beat per minute (Andersson, 2013), the impact was not significant. Furthermore, mirabegron is extensively metabolized by the liver and excreted in urine, either as the parent drug or its metabolites (Kashyap and Tyagi, 2013). Therefore, in clinical practice, it is not only important to pay attention to the safe dosage of mirabegron but also to monitor the liver and kidney functions of patients, avoiding excessively high blood drug concentrations that may lead to cardiovascular-related AEs. For patients experiencing cardiovascular-related AEs after using mirabegron, it is recommended to undergo blood drug concentration monitoring. Furthermore, this study also identified some unexpected yet clinically significant safety signals, such as angina pectoris, extrasystoles, and cardiac fibrillation. Although the reported cases of these cardiovascular AEs are limited, they should not be overlooked, as they may have potentially life-threatening implications for patients.

Evidence indicated that mirabegron could cause the AE of headache (Hou et al., 2021). In our study, we also identified head discomfort as a significant AE associated with mirabegron, with a signal strength of ROR 3.41 (1.28–9.08) and IC 1.77 (0.1). Additionally, in our results, dementia, transient ischemic attack, Parkinson’s disease, and myasthenia gravis emerged as new significant AEs associated with mirabegron. In a previous clinical study, 23,662 patients with OAB using mirabegron were found to have 603 new cases of dementia (Welk and McArthur, 2020). While the reported case numbers are limited, healthcare professionals should not overlook these findings, as mirabegron may potentially cause central nervous system side effects. In the striatum of both rats and humans, the presence of β-3 adrenergic receptor mRNA has been confirmed (Rodriguez et al., 1995). Activation of these receptors by mirabegron decreases acetylcholine release from striatal neurons (Murchison et al., 2016). Acetylcholine deficiency in the brain is linked not only to Alzheimer’s disease, vascular dementia, and Lewy body dementia but also to movement disorders (Sarter and Bruno, 1998; Jia et al., 2004; Murchison et al., 2016; Hoskin et al., 2019). Furthermore, β3-adrenergic receptor agonists promote the synthesis and release of serotonin (5-HT) in the striatum of the brain, which may also be an important mechanism (Murchison et al., 2016). In this study, we identified transient ischemic attack as an AE with significant signal strength, which was not previously listed in the drug label or observed in earlier clinical studies. As of now, the mechanism by which mirabegron induces transient ischemic attack remains unclear. Transient ischemic attack can be caused by various factors, including atherosclerosis, cardiac-related factors, arterial inflammation, and others. Atrial fibrillation was a known risk factor for transient ischemic attack (Thakur et al., 2023). Therefore, transient ischemic attack may also be associated with mirabegron-related AEs such as atrial fibrillation. The neurologic AEs caused by mirabegron were unexpected for clinicians, and if not promptly identified, they could potentially result in irreversible damage to the nervous system. Nevertheless, it is imperative to acknowledge that the incidence of dementia, transient ischemic attack, and Parkinson’s disease was subject to multifactorial influences, including lifestyle choices, age, genetic predisposition, and other variables. Consequently, prudent consideration of these findings is warranted.

Some other new and unexpected adverse events, including lip swelling, swollen tongue, mouth swelling, peptic ulcer, ischemic colitis, eyelid edema, glaucoma, and retinal vein occlusion, were also identified in this study. Matthew et al. (Zuchowski et al., 2022) reported a case of a patient who experienced tongue swelling, accompanied by difficulty breathing and swallowing, after using mirabegron. In this report, swelling was also observed in areas such as the lips, oral cavity, and eyelids. The mechanism of mirabegron-induced edema is believed to be a type I hypersensitivity reaction mediated by mast cell degranulation (Zuchowski et al., 2022).

It is noteworthy that both “anti-neutrophil cytoplasmic antibody increased” and “anti-neutrophil cytoplasmic antibody positive vasculitis” exhibited strong positive signals in both the ROR and IC methods. Anti-neutrophil cytoplasmic antibody positive vasculitis is a group of potentially life-threatening autoimmune diseases (Schönermarck et al., 2015; Sun et al., 2023). Although the exact etiology remains incompletely understood, research indicated that drugs are significant contributors to the development of this condition (Weng and Liu, 2019). Based on previous research findings, the drugs that induced anti-neutrophil cytoplasmic antibody positive vasculitis belonged to various pharmacological categories (Weng and Liu, 2019). These primarily included anti-thyroid drugs, tumor necrosis factor inhibitors, anti-tuberculosis drugs, psychoactive agents, and others (Weng and Liu, 2019). To the best of our knowledge, this study was the first to report the potential induction of anti-neutrophil cytoplasmic antibody positive vasculitis by mirabegron. The findings of this study merit careful attention from clinicians. Close monitoring of ANCA levels in patients undergoing mirabegron treatment is essential, as it serves as an effective tool for the early diagnosis of drug-induced anti-neutrophil cytoplasmic antibody positive vasculitis. Upon confirmation, immediate discontinuation of the drug is imperative, as most patients experience relief after discontinuing the use of this harmful medication (Weng and Liu, 2019).

Additionally, subgroup analysis revealed that AEs suggestive of hypertension and angioedema occurred in the group with a weight greater than 100 kg, which were absent in the group with a weight less than 50 kg. Research found that when the β3-adrenergic receptors in perivascular adipose tissue (PVAT) were stimulated, nitric oxide (NO) derived from adipocytes played an anticontractile role (Bussey et al., 2018). Additionally, individuals with lower functionality of β3-adrenergic receptors tended to experience weight gain (Andersson et al., 2009). This could also have been one of the potential mechanisms for hypertension in individuals with high body weight, but further research was still needed. Additionally, AEs related to mirabegron varied among different continent groups, which may be associated with polymorphisms of the β3-adrenergic receptor in different ethnicities. This merits further investigation. Aspirin and atorvastatin were commonly used medications for cardiovascular disease patients. Therefore, when analyzing AEs associated with the combination of mirabegron with aspirin or atorvastatin, significant signals were observed within the SOC category of cardiac disorders, such as atrial fibrillation, myocardial infarction, and hypertension.

The study findings revealed a median onset time of 25 days, with most AEs happening within the initial 30 days following exposure to mirabegron (n = 358, 55.68%). However, AEs could still occur up to a year later. Therefore, clinicians needed to closely follow up with patients who are using mirabegron, especially within the first 30 days. In future clinical studies, longer follow-up periods were necessary to observe mirabegron-related AEs.

Several limitations in this study need to be addressed. Firstly, the FAERs database is a spontaneous reporting database, and the quality is not rigorously controlled. Although our analysis included reports provided only by healthcare professionals to ensure the reliability of the reporting sources, it inevitably reduced the sample size. Additionally, the occurrence rates of each AE related to mirabegron could not be estimated. Secondly, the presence of reports in the FAERS database does not establish a causal relationship, requiring further well-designed clinical trials to investigate causation. Ultimately, certain confounding factors not measured in the study, such as possible interactions between medications, existing medical conditions, and combinations of drugs, were excluded from the analysis. Despite these limitations, FAERs remains valuable for post-marketing safety surveillance.

## Conclusion

In conclusion, despite numerous clinical studies confirming the efficacy and high safety profile of mirabegron in treating OAB, the increasing use of mirabegron necessitates clinicians to be aware of unexpected AEs not documented in the drug label or discovered in clinical trials. This study, which utilized a pharmacovigilance analysis of the FAERS database, scientifically elucidated the true safety profile of mirabegron treatment. New significant AEs, such as arrhythmia, palpitations, dementia, transient ischemic attack, Parkinson’s disease, anti-neutrophil cytoplasmic antibody positive vasculitis, lip swelling, swollen tongue, and others, were identified. Our research provided valuable evidence for further investigation and clinical practice involving mirabegron.

## Data Availability

The datasets presented in this study can be found in online repositories. The names of the repository/repositories and accession number(s) can be found below: https://www.fda.gov/drugs/questions-and-answers-fdas-adverse-event-reporting-systemfaers/fda-adverse-event-reporting-system-faers-public-dashboard.

## References

[B1] Andersson D. Wahrenberg H. Löfgren P. (2009). Beta3-adrenoceptor function and long-term changes in body weight. Int. J. Obes. 33, 662–668. 10.1038/ijo.2009.54 19365393

[B2] Andersson K. E. (2013). New developments in the management of overactive bladder: focus on mirabegron and onabotulinumtoxinA. Ther. Clin. Risk Manag. 9, 161–170. 10.2147/TCRM.S33052 23637536 PMC3634323

[B3] Balachandran A. A. Duckett J. R. (2015). The risk and severity of developing symptomatic palpitations when prescribed mirabegron for overactive bladder. Eur. J. Obstet. Gynecol. Reprod. Biol. 187, 60–63. 10.1016/j.ejogrb.2015.02.020 25756594

[B4] Batista J. E. Kölbl H. Herschorn S. Rechberger T. Cambronero J. Halaska M. (2015). The efficacy and safety of mirabegron compared with solifenacin in overactive bladder patients dissatisfied with previous antimuscarinic treatment due to lack of efficacy: results of a noninferiority, randomized, phase IIIb trial. Ther. Adv. Urol. 7 (4), 167–179. 10.1177/1756287215589250 26445596 PMC4580095

[B5] Bussey C. E. Withers S. B. Saxton S. N. Bodagh N. Aldous R. G. Heagerty A. M. (2018). β3 -Adrenoceptor stimulation of perivascular adipocytes leads to increased fat cell-derived NO and vascular relaxation in small arteries. Br. J. Pharmacol. 175, 3685–3698. 10.1111/bph.14433 29980164 PMC6109217

[B6] Chan C. S. Lin F. J. Liu C. M. Lin Y. K. Chen Y. C. Hsu C. C. (2022). Mirabegron, a β3-adrenoreceptor agonist, regulates right and left atrial arrhythmogenesis differently. Exp. Ther. Med. 24 (6), 720. 10.3892/etm.2022.11656 36340605 PMC9627121

[B7] Frey J. N. Vidal A. Krebs J. Christmann C. (2023). Percutaneous tibial nerve stimulation in the treatment of refractory idiopathic overactive bladder syndrome: a retrospective cohort study. J. Clin. Med. 12 (21), 6783. 10.3390/jcm12216783 37959248 PMC10648249

[B8] Haylen B. T. Maher C. F. Barber M. D. Camargo S. Dandolu V. Digesu A. (2016). An International Urogynecological Association (IUGA)/International Continence Society (ICS) joint report on the terminology for female pelvic organ prolapse (POP). Int. Urogynecol J. 27 (4), 655–684. 10.1007/s00192-016-3003-y 26984443

[B9] Herschorn S. Barkin J. Castro-Diaz D. Frankel J. M. Espuna-Pons M. Gousse A. E. (2013). A phase III, randomized, double-blind, parallel-group, placebo-controlled, multicentre study to assess the efficacy and safety of the β₃ adrenoceptor agonist, mirabegron, in patients with symptoms of overactive bladder. Urology 82 (2), 313–320. 10.1016/j.urology.2013.02.077 23769122

[B10] Hoskin J. L. Al-Hasan Y. Sabbagh M. N. (2019). Nicotinic acetylcholine receptor agonists for the treatment of Alzheimer's dementia: an update. Nicotine Tob. Res. 21 (3), 370–376. 10.1093/ntr/nty116 30137524 PMC6379052

[B11] Hou J. Xu F. Du H. Li N. (2021). Adverse events associated with mirabegron 50mg versus placebo: a systematic review and meta-analysis. Prog. Urol. 31 (11), 627–633. 10.1016/j.purol.2021.05.005 34312078

[B12] Javed F. Kumar A. (2024). Identification of signal of clindamycin associated renal failure acute: a disproportionality analysis. Curr. Drug Saf. 19, 123–128. 10.2174/1574886318666230228142856 36852785

[B13] Ji X. Wang L. Hua L. Wang X. Zhang P. Shendre A. (2021). Improved adverse drug event prediction through information component guided pharmacological Network model (IC-pnm). IEEE/ACM Trans. Comput. Biol. Bioinform 18, 1113–1121. 10.1109/TCBB.2019.2928305 31443040

[B14] Jia J. P. Jia J. M. Zhou W. D. Xu M. Chu C. b. Yan X. (2004). Differential acetylcholine and choline concentrations in the cerebrospinal fluid of patients with Alzheimer's disease and vascular dementia. Chin. Med. J. Engl. 117 (8), 1161–1164. 15361288

[B15] Kashyap M. Tyagi P. (2013). The pharmacokinetic evaluation of mirabegron as an overactive bladder therapy option. Expert Opin. Drug Metab. Toxicol. 9 (5), 617–627. 10.1517/17425255.2013.786700 23550899

[B16] Korstanje C. Suzuki M. Yuno K. Sato S. Ukai M. Schneidkraut M. J. (2017). Translational science approach for assessment of cardiovascular effects and proarrhythmogenic potential of the beta-3 adrenergic agonist mirabegron. J. Pharmacol. Toxicol. Methods 87, 74–81. 10.1016/j.vascn.2017.04.008 28434969

[B17] Krhut J. Wohlfahrt P. Pudich J. Kufová E. Borovička V. Bílková K. (2021). Cardiovascular safety of mirabegron in individuals treated for spinal cord injury- or multiple sclerosis-induced neurogenic detrusor overactivity. Int. Urol. Nephrol. 53 (6), 1089–1095. 10.1007/s11255-020-02774-7 33417146

[B18] Kuo H. C. (2022). How to choose appropriate medication for overactive bladder: findings from the largest integrated clinical trial database analysis of mirabegron studies. Tzu Chi Med. J. 34, 23–28. 10.4103/tcmj.tcmj_167_20 35233352 PMC8830553

[B19] Kuo Y. C. Kuo H. C. (2023). Comparative study of different combinations of mirabegron and antimuscarinics in treatment for overactive bladder syndrome in elderly patients. Tzu Chi Med. J. 35 (1), 62–68. 10.4103/tcmj.tcmj_209_21 36866344 PMC9972936

[B20] Lu Z. Zhang J. Lin S. Fan Z. He Z. Tang F. (2023). Associations between overactive bladder and sleep patterns: a cross-sectional study based on 2007-2014 NHANES. BMC Urol. 23 (1), 184. 10.1186/s12894-023-01329-z 37957629 PMC10642019

[B21] Malik M. van Gelderen E. M. Lee J. H. Kowalski D. L. Yen M. Goldwater R. (2012). Proarrhythmic safety of repeat doses of mirabegron in healthy subjects: a randomized, double-blind, placebo-and active-controlled thorough QT study. Clin. Pharmacol. Ther. 92 (6), 696–706. 10.1038/clpt.2012.181 23149929

[B22] Malsin E. S. Coleman J. M. Wolfe L. F. Lam A. P. (2019). Respiratory dysfunction following initiation of mirabegron: a case report. Respir. Med. Case Rep. 26, 304–306. 10.1016/j.rmcr.2019.02.012 30886821 PMC6402286

[B23] Murchison A. G. Fletcher C. Cheeran B. (2016). Recurrence of dyskinesia as a side-effect of mirabegron in a patient with Parkinson's disease on DBS (GPi). Park. Relat. Disord. 27, 107–108. 10.1016/j.parkreldis.2016.03.009 27004469

[B24] Nitti V. W. Auerbach S. Martin N. Calhoun A. Lee M. Herschorn S. (2013a). Results of a randomized phase III trial of mirabegron in patients with overactive bladder. J. Urol. 189 (4), 1388–1395. 10.1016/j.juro.2012.10.017 23079373

[B25] Nitti V. W. Khullar V. van Kerrebroeck P. Herschorn S. Cambronero J. Angulo J. C. (2013b). Mirabegron for the treatment of overactive bladder: a prespecified pooled efficacy analysis and pooled safety analysis of three randomised, double-blind, placebo-controlled, phase III studies. Int. J. Clin. Pract. 67 (7), 619–632. 10.1111/ijcp.12194 23692526 PMC3752932

[B26] Nitti V. W. Rosenberg S. Mitcheson D. H. He W. Fakhoury A. Martin N. E. (2013c). Urodynamics and safety of the β₃-adrenoceptor agonist mirabegron in males with lower urinary tract symptoms and bladder outlet obstruction. J. Urol. 190 (4), 1320–1327. 10.1016/j.juro.2013.05.062 23727415

[B27] Noguchi Y. Yoshizawa S. Aoyama K. Kubo S. Tachi T. Teramachi H. (2021). Verification of the "upward variation in the reporting odds ratio scores" to detect the signals of drug-drug interactions. Pharmaceutics 13, 1531. 10.3390/pharmaceutics13101531 34683823 PMC8537362

[B28] Passos G. R. de Oliveira M. G. Ghezzi A. C. Mello G. C. Levi D'Ancona C. A. Teixeira S. A. (2023). Periprostatic adipose tissue (PPAT) supernatant from obese mice releases anticontractile substances and increases human prostate epithelial cell proliferation: the role of nitric oxide and adenosine. Front. Pharmacol. 14, 1145860. 10.3389/fphar.2023.1145860 37492091 PMC10364323

[B29] Rodriguez M. Carillon C. Coquerel A. Le Fur G. Ferrara P. Caput D. (1995). Evidence for the presence of beta 3-adrenergic receptor mRNA in the human brain. Brain Res. Mol. Brain Res. 29 (2), 369–375. 10.1016/0169-328x(94)00274-i 7609625

[B30] Rosa G. M. Ferrero S. Nitti V. W. Wagg A. Saleem T. Chapple C. R. (2016). Cardiovascular safety of β3-adrenoceptor agonists for the treatment of patients with overactive bladder syndrome. Eur. Urol. 69 (2), 311–323. 10.1016/j.eururo.2015.09.007 26422675

[B31] Rothman K. J. Lanes S. Sacks S. T. (2004). The reporting odds ratio and its advantages over the proportional reporting ratio. Pharmacoepidemiol Drug Saf. 13, 519–523. 10.1002/pds.1001 15317031

[B32] Sarter M. Bruno J. P. (1998). Cortical acetylcholine, reality distortion, schizophrenia, and Lewy Body Dementia: too much or too little cortical acetylcholine. Brain Cogn. 38 (3), 297–316. 10.1006/brcg.1998.1035 9841788

[B33] Sartori L. Nunes B. M. Farah D. Oliveira L. M. d. Novoa C. C. T. Sartori M. G. F. (2023). Mirabegron and anticholinergics in the treatment of overactive bladder syndrome: a meta-analysis. Rev. Bras. Ginecol. Obstet. 45 (6), 337–346. 10.1055/s-0043-1770093 37494577 PMC10371066

[B34] Schönermarck U. Csernok E. Gross W. L. (2015). Pathogenesis of anti-neutrophil cytoplasmic antibody-associated vasculitis: challenges and solutions 2014. Nephrol. Dial. Transpl. 30 (1), i46–i52. 10.1093/ndt/gfu398 25540095

[B35] Sharma A. Kumar A. (2022). Identification of novel signal of clobazam-associated drug reaction with eosinophilia and systemic symptoms syndrome: a disproportionality analysis. Acta Neurol. Scand. 146, 623–627. 10.1111/ane.13690 36029138

[B36] Shu Y. He X. Liu Y. Wu P. Zhang Q. (2022). A real-world disproportionality analysis of olaparib: data mining of the public version of FDA adverse event reporting system. Clin. Epidemiol. 14, 789–802. 10.2147/CLEP.S365513 35789689 PMC9250344

[B37] Silva I. Costa A. F. Moreira S. Ferreirinha F. Magalhães-Cardoso M. T. Calejo I. (2017). Inhibition of cholinergic neurotransmission by β3-adrenoceptors depends on adenosine release and A1-receptor activation in human and rat urinary bladders. Am. J. Physiol. Ren. Physiol. 313, F388–F403. 10.1152/ajprenal.00392.2016 28446460

[B38] SongW.HuH.NiJ.ZhangH.ZhangY.ZhangH. (2023). The role of sarcopenia in overactive bladder in adults in the United States: retrospective analysis of NHANES 2011-2018. J. Nutr. Health Aging 27 (9), 734–740. 10.1007/s12603-023-1972-3 37754213

[B39] Stewart W. F. Van Rooyen J. B. Cundiff G. W. Abrams P. Herzog A. R. Corey R. (2003). Prevalence and burden of overactive bladder in the United States. World J. Urol. 20 (6), 327–336. 10.1007/s00345-002-0301-4 12811491

[B40] Sun X. J. Li Z. Y. Chen M. (2023). Pathogenesis of anti-neutrophil cytoplasmic antibody-associated vasculitis. Rheumatol. Immunol. Res. 4 (1), 11–21. 10.2478/rir-2023-0003 37138650 PMC10150877

[B41] Takahashi S. Kato D. Tabuchi H. Uno S. (2021). Safety and effectiveness of mirabegron in male patients with overactive bladder with or without benign prostatic hyperplasia: a Japanese post-marketing study. Low. Urin Tract. Symptoms 13 (1), 79–87. 10.1111/luts.12335 32761776 PMC7818393

[B42] Tan M. G. Burns B. F. Glassman S. J. (2019). Serum sickness-like reaction associated with mirabegron. JAAD Case Rep. 5 (6), 537–539. 10.1016/j.jdcr.2019.04.010 31205999 PMC6558296

[B43] Thakur M. Alsinbili A. Chattopadhyay R. Warburton E. A. Khadjooi K. Induruwa I. (2023). Identifying the optimal time period for detection of atrial fibrillation after ischaemic stroke and TIA: an updated systematic review and meta-analysis of randomized control trials. Int. J. Stroke, 17474930231215277. 10.1177/17474930231215277 37947341

[B44] van Gelderen M. Stölzel M. Meijer J. Kerbusch V. Collins C. Korstanje C. (2017). An exploratory study in healthy male subjects of the mechanism of mirabegron-induced cardiovascular effects. J. Clin. Pharmacol. 57 (12), 1534–1544. 10.1002/jcph.952 28618007

[B45] Welk B. McArthur E. (2020). Increased risk of dementia among patients with overactive bladder treated with an anticholinergic medication compared to a beta-3 agonist: a population-based cohort study. BJU Int. 126 (1), 183–190. 10.1111/bju.15040 32167223

[B46] Weng C. H. Liu Z. C. (2019). Drug-induced anti-neutrophil cytoplasmic antibody-associated vasculitis. Chin. Med. J. Engl. 132 (23), 2848–2855. 10.1097/CM9.0000000000000539 31856057 PMC6940077

[B47] Xiao Y. Yin S. Wang J. Cui J. Yang Z. Wang J. (2023). A positive association between the prevalence of circadian syndrome and overactive bladder in United States adults. Front. Public Health 11, 1137191. 10.3389/fpubh.2023.1137191 37637821 PMC10449362

[B48] Yamaguchi O. Marui E. Igawa Y. Takeda M. Nishizawa O. Ikeda Y. (2015). Efficacy and safety of the selective β3 -adrenoceptor agonist mirabegron in Japanese patients with overactive bladder: a randomized, double-blind, placebo-controlled, dose-finding study. Low. Urin Tract. Symptoms 7 (2), 84–92. 10.1111/luts.12053 26663687

[B49] Zhang M. Xie W. Li J. Zheng J. Zhou Y. (2023). Postmarketing safety profile of brexanolone: a pharmacovigilance analysis based on FDA Adverse Event Reporting System (FAERS). Arch. Womens Ment. Health 27, 35–44. 10.1007/s00737-023-01378-1 37831172

[B50] Zuchowski M. T. Yuksel J. M. Novi J. (2022). Mirabegron associated angioedema: a case report. Hosp. Pharm. 57 (6), 771–773. 10.1177/00185787221095737 36340626 PMC9631014

