# Supplementary material for: Examining the safety of mirabegron: an analysis of real-world pharmacovigilance data from the US FDA adverse event reporting system (FAERS) database
Source: Front Pharmacol. 2024 Mar 18;15:1376535. doi: 10.3389/fphar.2024.1376535 (PMC10982368; doi:10.3389/fphar.2024.1376535)
Supplement: Supplementary file 1 [file DataSheet1.pdf]

Subgroup	PT (Perferred terms)	N	ROR(95% CI)
< 50 kg	Urinary retention	5	95.86(38.56, 238.29)
	Tachycardia	3	12.62(3.98, 40.02)
50 ~ 100 kg	Cystitis haemorrhagic	3	63.64(20.33, 199.29)
	Muscle atrophy	3	23.93(7.68, 74.59)
	Micturition urgency	3	23.66(7.59, 73.75)
	Muscle tightness	3	20.94(6.72, 65.25)
	Heart rate irregular	4	19.31(7.21, 51.69)
	Kidney infection	3	19.07(6.12, 59.42)
	Cystitis	5	12.64(5.24, 30.50)
	Arrhythmia	7	11.72(5.56, 24.70)
	Urinary retention	6	11.63(5.20, 26.02)
	Dysuria	6	11.61(5.19, 25.96)
	Haematuria	7	10.20(4.84, 21.49)
	Liver disorder	5	8.66(3.59, 20.88)
	Urinary incontinence	3	7.73(2.48, 24.04)
	Dry mouth	5	7.26(3.01, 17.50)
	Pollakiuria	3	7.23(2.32, 22.49)
	Blood pressure increased	10	7.07(3.78, 13.20)
	Electrocardiogram qt prolonged	4	6.27(2.34, 16.76)
	Atrial fibrillation	10	5.68(3.04, 10.61)
	Cardiac failure	8	5.64(2.81, 11.33)
	Palpitations	8	5.64(2.81, 11.33)
> 100 kg	Nocturia	3	54.33(17.17, 171.86)
	Urinary retention	5	46.06(18.81, 112.77)
	Urinary incontinence	3	29.04(9.23, 91.41)
	Atrial fibrillation	7	9.94(4.67, 21.18)
	Hypertension	6	7.59(3.36, 17.14)
	Angioedema	3	4.41(1.41, 13.83)

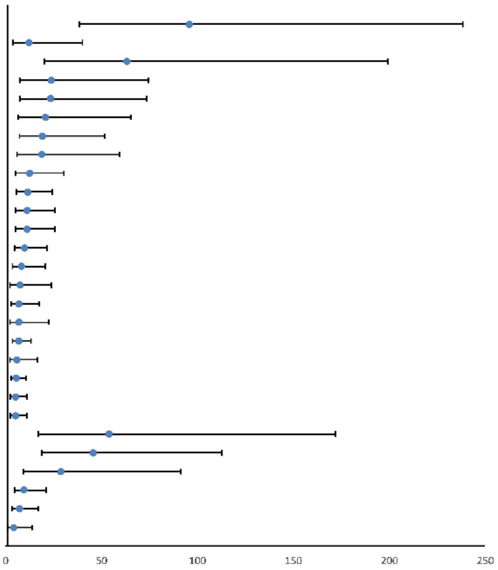

Supplemental Figure S1 The top 20 preferred terms level adverse events for mirabegron in different weight groups, ranked by signal strength in the Food and Drug Administration (FDA) Adverse Event Reporting System (FAERS).

Subgroup	PT (Perferred terms)	N	ROR(95% CI)
North America	Antineutrophil cytoplasmic antibody increased	3	335.49(101.94,1104.17)
	Urge incontinence	3	83.87(26.63,264.16)
	Urinary retention	80	74.57(59.56,93.36)
	Nocturia	28	73.09(50.16,106.51)
	Bladder pain	6	57.69(25.71,129.46)
	Urine flow decreased	4	56.18(20.89,151.12)
	Cystitis interstitial	3	50.50(16.13,158.12)
	Bladder spasm	3	32.17(10.31,100.39)
	Hypertensive crisis	6	26.83(12.00,59.97)
	Micturition urgency	10	21.98(11.79,40.98)
	Blood pressure increased	126	20.70(17.31,24.77)
	Urinary incontinence	18	16.41(10.31,26.11)
	Dry mouth	34	11.93(8.50,16.73)
	Blood pressure systolic increased	10	11.15(5.99,20.77)
	Dry throat	4	10.67(4.00,28.50)
	Hypertension	72	8.76(6.93,11.08)
	Parkinson's disease	5	8.58(3.56,20.65)
	Pollakiuria	13	8.09(4.69,13.97)
	Glaucoma	5	7.97(3.31,19.18)
	Dysuria	10	7.19(3.86,13.39)
Europe	Accelerated hypertension	5	175.92(70.15,441.18)
	Bladder pain	4	70.34(25.88,191.17)
	Malignant hypertension	5	70.09(28.66,171.44)
	Hypertonic bladder	3	61.82(19.54,195.57)
	Essential hypertension	3	42.69(13.57,134.23)
	Cardiac fibrillation	4	41.47(15.38,111.84)
	Cardiac discomfort	3	39.55(12.59,124.24)
	Heart rate irregular	10	27.88(14.91,52.13)
	Micturition urgency	6	19.40(8.67,43.43)
	Mouth swelling	5	15.49(6.42,37.40)
	Hypertensive crisis	27	12.85(8.78,18.81)
	Kidney infection	3	12.28(3.94,38.25)
	Extrasystoles	5	12.02(4.99,29.00)
	Head discomfort	4	11.35(4.24,30.36)
	Pollakiuria	9	11.16(5.79,21.52)
	Atrial fibrillation	65	10.52(8.22,13.48)
	Palpitations	43	10.45(7.72,14.15)
	Muscle tightness	3	10.38(3.33,32.32)
	Arrhythmia	30	10.26(7.15,14.72)
	Urinary retention	23	9.76(6.46,14.73)
Asia	Peptic ulcer	4	40.65(15.00,110.10)
	Arrhythmia	49	37.63(28.13,50.36)
	Erectile dysfunction	4	31.35(11.61,84.65)
	Postrenal failure	4	29.03(10.76,78.32)
	Urinary retention	24	20.42(13.58,30.72)
	Benign prostatic hyperplasia	3	20.25(6.46,63.43)
	Retinal vein occlusion	4	19.39(7.21,52.12)
	Dysuria	9	17.28(8.93,33.45)
	Sinus node dysfunction	4	16.28(6.06,43.72)
	Dry mouth	4	14.48(5.39,38.85)
	Cystitis	4	11.36(4.24,30.45)
	Cognitive disorder	6	10.96(4.90,24.55)
	Electrocardiogram qt prolonged	16	10.63(6.47,17.46)
	Dementia	9	10.00(5.18,19.33)
	Ventricular extrasystoles	3	9.79(3.14,30.52)
	Blood pressure increased	14	9.61(5.66,16.32)
	Pollakiuria	3	9.56(3.06,29.81)
	Prostate cancer	7	9.55(4.53,20.14)
	Atrioventricular block complete	3	9.15(2.94,28.54)
	Dyspepsia	3	7.77(2.49,24.21)

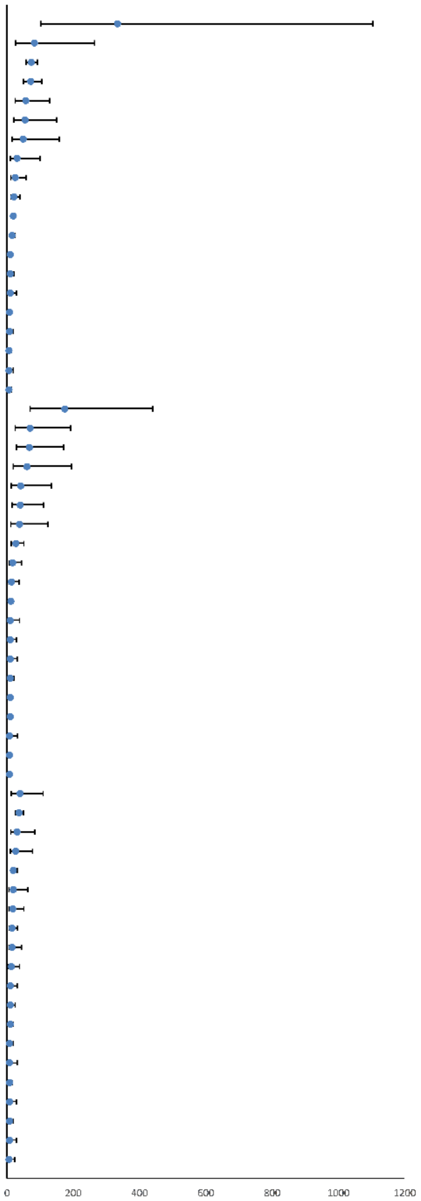

Supplemental Figure S2 The top 20 preferred terms level adverse events for mirabegron in different continent groups, ranked by signal strength in the Food and Drug Administration (FDA) Adverse Event Reporting System (FAERS).

Subgroup	PT (Perferred terms)	N	ROR(95% CI)
Mirabegron monotherapy	Antineutrophil cytoplasmic antibody increased	5	170.08(68.92,419.68)
	Accelerated hypertension	4	80.83(29.90,218.51)
	Bladder pain	10	57.32(30.63,107.28)
	Cystitis interstitial	4	40.27(15.00,108.10)
	Urine flow decreased	5	37.29(15.42,90.15)
	Nocturia	27	34.46(23.55,50.40)
	Cardiac fibrillation	4	34.01(12.68,91.19)
	Urinary retention	121	33.33(27.81,39.95)
	Hypertonic bladder	7	32.55(15.44,68.61)
	Malignant hypertension	4	32.24(12.03,86.42)
	Postrenal failure	3	31.45(10.07,98.16)
	Lip disorder	3	24.18(7.76,75.35)
	Cardiac discomfort	3	21.61(6.94,67.33)
	Micturition urgency	15	18.40(11.07,30.60)
	Arrhythmia	81	17.72(14.22,22.08)
	Hypertensive crisis	32	17.36(12.25,24.59)
	Blood pressure increased	157	15.29(13.04,17.93)
	Extrasystoles	8	13.88(6.93,27.82)
	Vulvovaginal discomfort	3	13.56(4.36,42.16)
	Neurogenic bladder	3	13.03(4.19,40.52)
Mirabegron combined with aspirin	Ventricular extrasystoles	3	28.36(9.11,88.26)
	Urinary retention	7	19.81(9.39,41.76)
	Atrial fibrillation	15	14.33(8.58,23.95)
	Palpitations	9	10.67(5.52,20.62)
	Swollen tongue	3	10.61(3.41,32.99)
	Tachycardia	10	9.27(4.96,17.34)
	Blood pressure increased	9	9.03(4.67,17.46)
	Dry mouth	4	8.47(3.17,22.66)
	Haematuria	3	6.75(2.17,20.99)
	Acute myocardial infarction	3	6.72(2.16,20.89)
	Hypertension	11	6.21(3.42,11.28)
	Epistaxis	4	5.74(2.15,15.36)
	Electrocardiogram qt prolonged	3	5.24(1.68,16.29)
	Chest pain	8	5.19(2.58,10.43)
	Bradycardia	3	3.79(1.22,11.78)
	Myocardial infarction	5	3.69(1.53,8.91)
	Syncope	4	3.64(1.36,9.74)

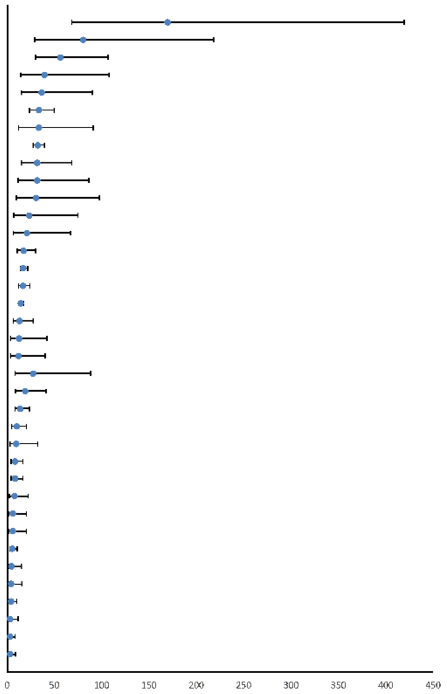

Supplemental Figure S3 The top 20 preferred terms level adverse events for mirabegron with or without concomitant use of aspirin, ranked by signal strength in the Food and Drug Administration (FDA) Adverse Event Reporting System (FAERS).

Subgroup	PT (Perferred terms)	N	ROR(95% CI)
Mirabegron monotherapy	Antineutrophil cytoplasmic antibody increased	5	164.09(66.50,404.91)
	Accelerated hypertension	3	58.06(18.49,182.28)
	Bladder pain	10	55.30(29.55,103.50)
	Malignant hypertension	5	39.00(16.12,94.33)
	Cystitis interstitial	4	38.85(14.48,104.29)
	Urine flow decreased	5	35.98(14.88,86.98)
	Nocturia	27	33.24(22.72,48.62)
	Cardiac fibrillation	4	32.81(12.24,87.98)
	Urinary retention	123	32.68(27.31,39.11)
	Urge incontinence	3	30.69(9.83,95.79)
	Postrenal failure	3	30.34(9.72,94.70)
	Hypertonic bladder	6	26.87(12.02,60.07)
	Lip disorder	3	23.33(7.48,72.70)
	Bladder spasm	3	20.96(6.73,65.30)
	Cardiac discomfort	3	20.85(6.69,64.96)
	Micturition urgency	16	18.95(11.58,31.01)
	Hypertensive crisis	33	17.27(12.25,24.34)
	Arrhythmia	81	17.09(13.71,21.29)
	Blood pressure increased	161	15.13(12.93,17.70)
	Extrasystoles	8	13.39(6.68,26.84)
Mirabegron combined with atorvastatin	Erectile dysfunction	5	49.08(20.28,118.76)
	Heart rate irregular	3	35.86(11.50,111.82)
	Urinary retention	5	22.92(9.47,55.44)
	Atrial fibrillation	8	12.31(6.10,24.82)
	Hyperglycaemia	3	12.13(3.89,37.81)
	Palpitations	5	9.56(3.95,23.14)
	Electrocardiogram qt prolonged	3	8.50(2.73,26.48)
	Blood pressure increased	5	8.10(3.35,19.59)
	Vision blurred	4	7.37(2.75,19.76)
	Visual impairment	3	6.41(2.06,19.98)
	Chest pain	6	6.31(2.81,14.15)
	Hypertension	6	5.46(2.44,12.25)
	Cerebrovascular accident	4	4.94(1.84,13.25)
	Tachycardia	3	4.45(1.43,13.88)
	Oedema peripheral	3	4.12(1.32,12.83)
	Feeling abnormal	3	3.77(1.21,11.74)
	Myocardial infarction	3	3.58(1.15,11.16)

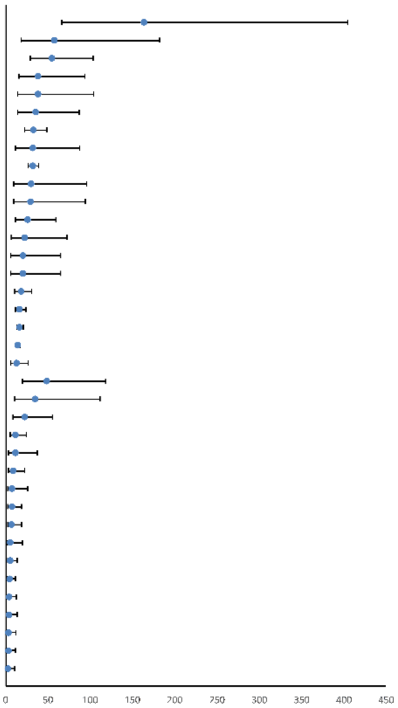

Supplemental Figure S4 The top 20 preferred terms level adverse events for mirabegron with or without concomitant use of atorvastatin, ranked by signal strength in the Food and Drug Administration (FDA) Adverse Event Reporting System (FAERS).

Subgroup	PT (Perferred terms)	N	ROR(95% CI)
Mirabegron monotherapy	Antineutrophil cytoplasmic antibody increased	5	163.15(66.12,402.57)
	Accelerated hypertension	5	97.65(39.99,238.44)
	Bladder pain	10	54.99(29.38,102.90)
	Postrenal failure	4	40.38(15.04,108.42)
	Malignant hypertension	5	38.78(16.03,93.79)
	Cystitis interstitial	4	38.63(14.39,103.69)
	Nocturia	29	35.54(24.61,51.31)
	Cardiac fibrillation	4	32.62(12.17,87.47)
	Urinary retention	123	32.49(27.15,38.88)
	Urge incontinence	3	30.51(9.77,95.24)
	Urine flow decreased	4	28.53(10.65,76.45)
	Lip disorder	3	23.19(7.44,72.28)
	Hypertonic bladder	5	22.22(9.21,53.60)
	Cardiac discomfort	3	20.73(6.66,64.59)
	Hypertensive crisis	34	17.70(12.62,24.82)
	Micturition urgency	15	17.65(10.62,29.35)
	Arrhythmia	82	17.20(13.82,21.41)
	Blood pressure increased	164	15.33(13.12,17.91)
	Extrasystoles	8	13.32(6.65,26.69)
	Vulvovaginal discomfort	3	13.00(4.18,40.44)
Mirabegron combined with solifenacin	Dry mouth	8	31.12(15.41,62.83)
	Urinary retention	5	25.56(10.56,61.87)
	Dry eye	4	21.78(8.12,58.43)
	Palpitations	7	15.04(7.11,31.82)
	Acute myocardial infarction	3	12.14(3.89,37.87)
	Delirium	3	12.09(3.88,37.70)
	Constipation	9	9.85(5.07,19.12)
	Visual impairment	3	7.14(2.29,22.28)
	Atrial fibrillation	4	6.78(2.53,18.18)
	Tachycardia	4	6.64(2.48,17.81)
	Dysphagia	3	5.92(1.90,18.47)
	Hypertension	5	5.06(2.09,12.25)
	Urinary tract infection	3	3.53(1.13,11.02)
	Insomnia	3	3.37(1.08,10.50)

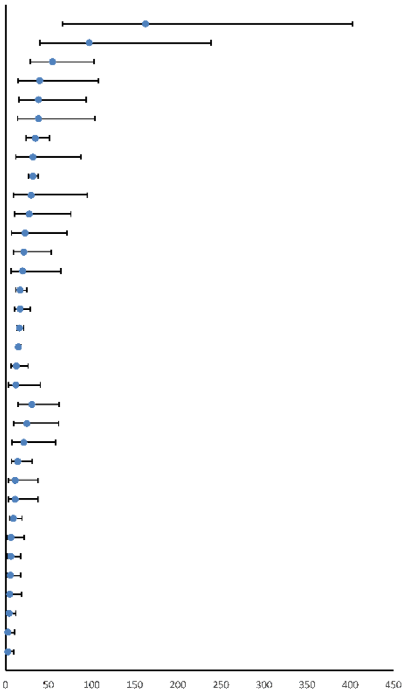

Supplemental Figure S5 The top 20 preferred terms level adverse events for mirabegron with or without concomitant use of solifenacin, ranked by signal strength in the Food and Drug Administration (FDA) Adverse Event Reporting System (FAERS).
